# Targeting the Tumor: Assessing the Impact of Bladder Volume and Position on Accuracy of Radiation Delivery for Patients with Bladder Cancer

**DOI:** 10.7759/cureus.1638

**Published:** 2017-09-01

**Authors:** Andrew Kochan, Ryan Rivest, Katie Galloway, Pascal Lambert, Aldrich Ong, Rashmi Koul, Shahida Ahmed, Bashir Bashir, Harvey Quon

**Affiliations:** 1 Faculty of Medicine, University of Manitoba; 2 Radiation Oncology, CancerCare Manitoba, University of Manitoba; 3 Epidemiology and Cancer Registry, CancerCare Manitoba, University of Manitoba; 4 Radiation Oncology, Tom Baker Cancer Centre

**Keywords:** cone-beam computed tomography, image-guided radiotherapy, organ size, regression analysis, urinary bladder neoplasms/radiotherapy

## Abstract

Context

Daily variations in bladder size and position can negatively impact the ability to accurately deliver radiation.

Aims

We attempted to quantify how bladder volumes and positions change over the course of radiotherapy for muscle invasive bladder cancer and the planning target volume (PTV) margins required to account for such changes.

Methods and material

Cone-beam computed tomography (CT) images of 28 patients during their first, second, and third fractions and weekly thereafter were acquired. Bladders were contoured and the volume, centre of mass, and the maximal positions were recorded and compared to the planning CT scan.

Statistical analysis

Bladder parameters were analysed using regression analysis examining for time trends and correlation to the patient, tumour, or treatment-related factors.

Results

There was great variability in the mean bladder volumes during the radiotherapy courses (154.17 +/- 129.38 cm^3^). There were no statistically significant trends for volume changes. Deviations in bladder positions were seen but were small in magnitude. No patient factors were identified which could help predict bladder changes clinically. Bladder variability resulted in a high percentage of fractions (39.6%) in which part of the bladder was outside the PTV. Calculated PTV margins (for 90% of the population to receive 95% of the prescription dose) were 1.48 cm right, 1.15 cm left, 2.13 cm posterior, 1.52 cm anterior, 2.23 cm superior, and 0.52 cm inferior.

Conclusions

Because of random bladder changes, a significant number of fractions were treated in which the clinical target volume (CTV) fell outside of the PTV. Methods to minimize the amount of CTV that is missed on a fraction to fraction basis should be explored.

## Introduction

The traditional treatment for muscle invasive bladder cancer has been radical cystectomy. Recent advances in bladder sparing chemoradiotherapy offer comparable outcomes to those of radical cystectomy [1-2]. However, daily variations in bladder size and position due to differences in bladder filling and other factors, such as the degree of rectal filling and bowel/prostate/uterus position, can negatively impact the ability to accurately deliver radiation to the bladder [3-4]. As a result, larger planning target volume (PTV) margins may be used. This increases the volume of organs at risk being irradiated, which can contribute to faecal urgency, incontinence, diarrhoea, and more serious complications, such as bowel strictures, fistulas, and contractures [5-7].

In this study, we attempted to quantify changes in bladder volume and position over the course of radiation therapy. We assessed the impact of patient factors (age, gender, body mass index (BMI), and disease stage) on daily variations in bladder position and size. We also determined the amount of bladder that extended outside the PTV in each image assessed. Then, we calculated the PTV margins for radiation treatment of muscle invasive bladder cancer. 

## Materials and methods

Between October 2013 and June 2015, 34 patients received radiation treatment for muscle invasive bladder cancer at our institution. Each patient initially underwent a planning computed tomography (CT) scan acquired on a Brilliance CT - Big Bore configuration system (Philips Healthcare, Amsterdam, The Netherlands). This scan was taken with the patient in the treatment position (lying supine with a leg rest under their knees). Planning CT scan images consisted of slices of 0.30 cm thickness and 1.17 mm pixel spacing. Images were then transferred to the Eclipse™ treatment planning software (Varian Medical Systems, Palo Alto, CA). Treatment was performed using a Varian Medical Systems Trilogy^®^ Series linear accelerator equipped with an On-Board Imager (OBI v1.5) (Varian Medical Systems, Palo Alto, CA) with the ability to take kilovoltage cone beam computed tomography (CBCT) images. The CBCT images consisted of 64 planes with 0.25 cm of separation and pixel spacing of 1.17 mm. Our routine practice is to acquire CBCT images of each patient during their first, second, and third fractions and weekly thereafter until the end of their treatment. The CBCT scans were taken with the patients in the treatment position, after alignment and before treatment. Patient alignment was performed using bony landmarks on orthogonal radiographic images. All patients were instructed to empty their bladder prior to the planning CT as well as the daily treatments.

**Figure 1:**
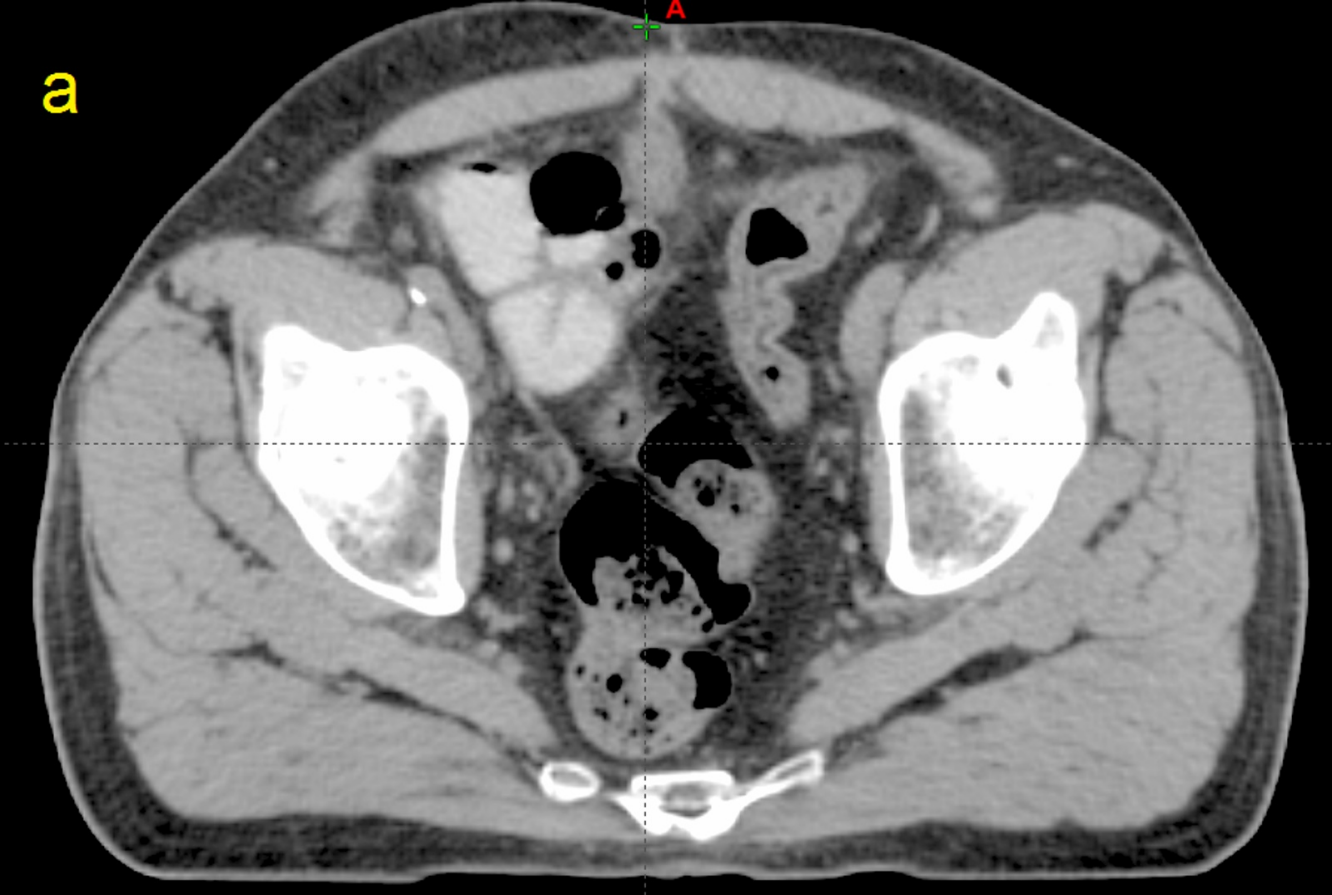
Computed tomographic (CT) image taken from patient P12 a) A slice from the planning CT scan.

**Figure 2:**
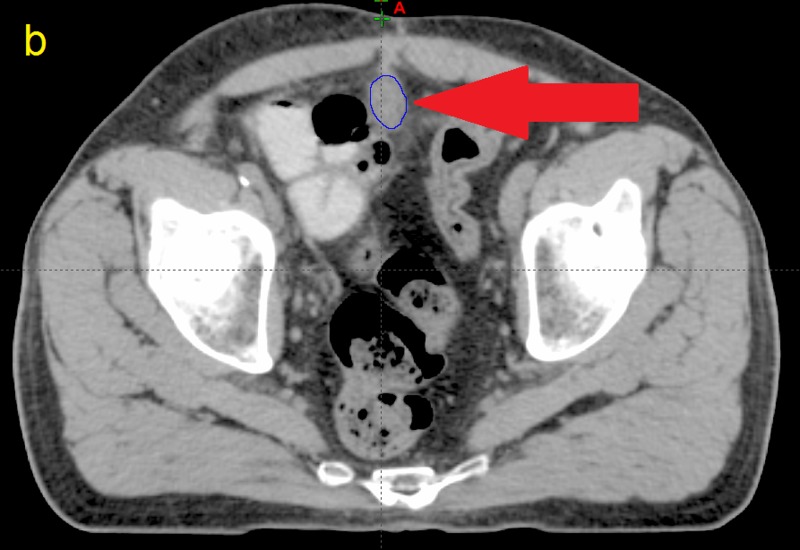
Computed tomographic (CT) image taken from patient P12 b) The same slice after contouring (blue)

**Figure 3:**
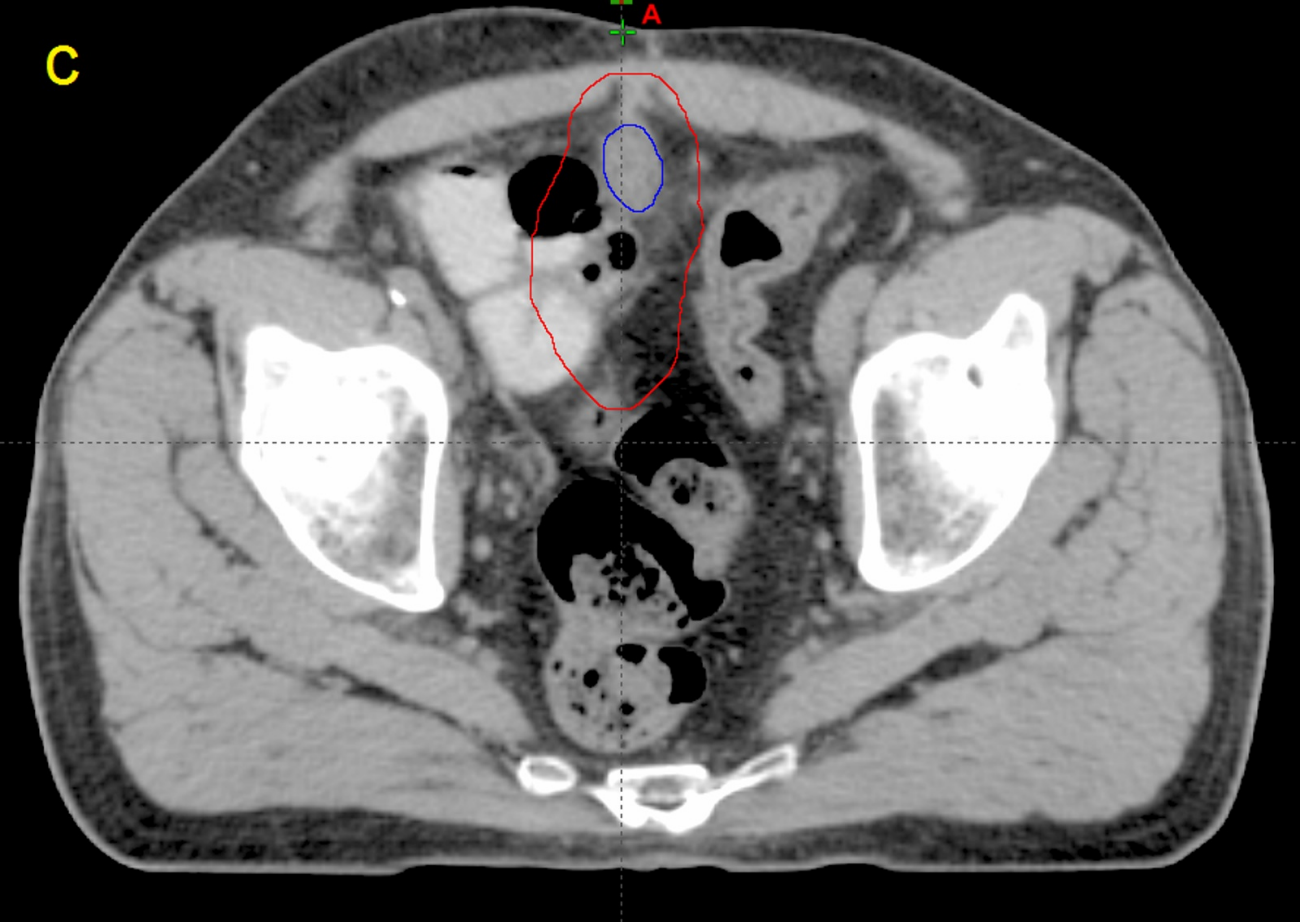
Computed tomographic (CT) image taken from patient P12 c) The planning target volume (red) generated from the contour

**Figure 4:**
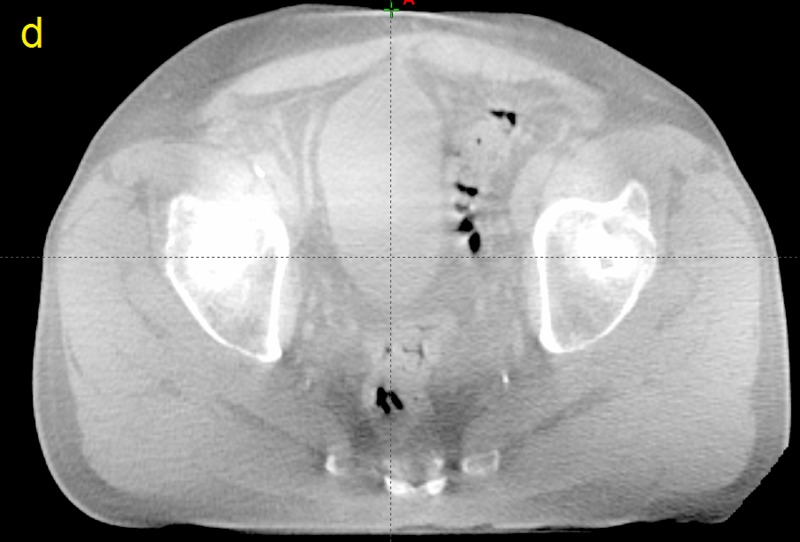
Computed tomographic (CT) image taken from patient P12 d) A slice taken from the cone beam computed tomography of fraction 2 at the same level as the previous images.

**Figure 5:**
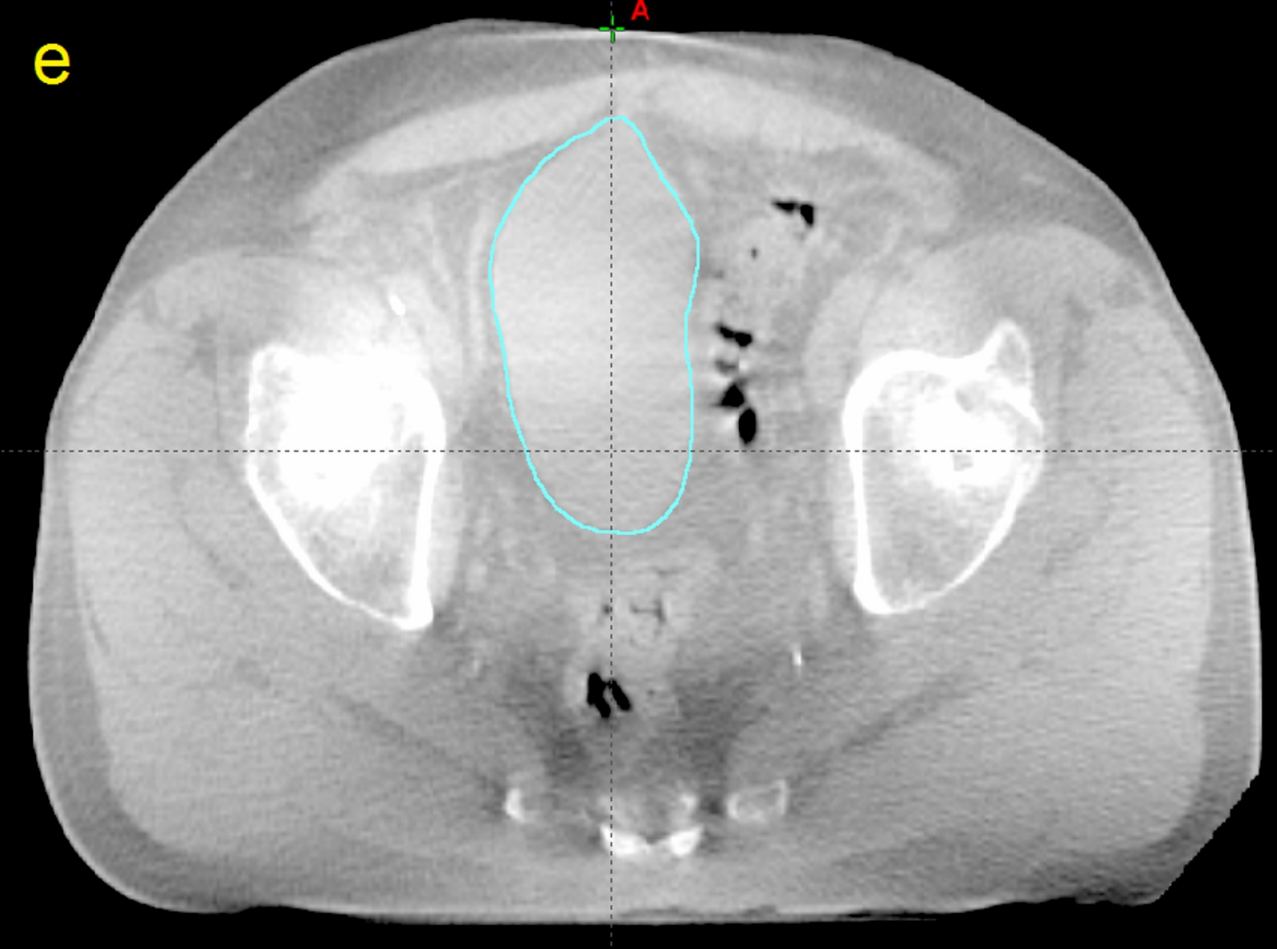
Computed tomographic (CT) image taken from patient P12 e) The cone beam computed tomography slice after contouring (teal).

**Figure 6:**
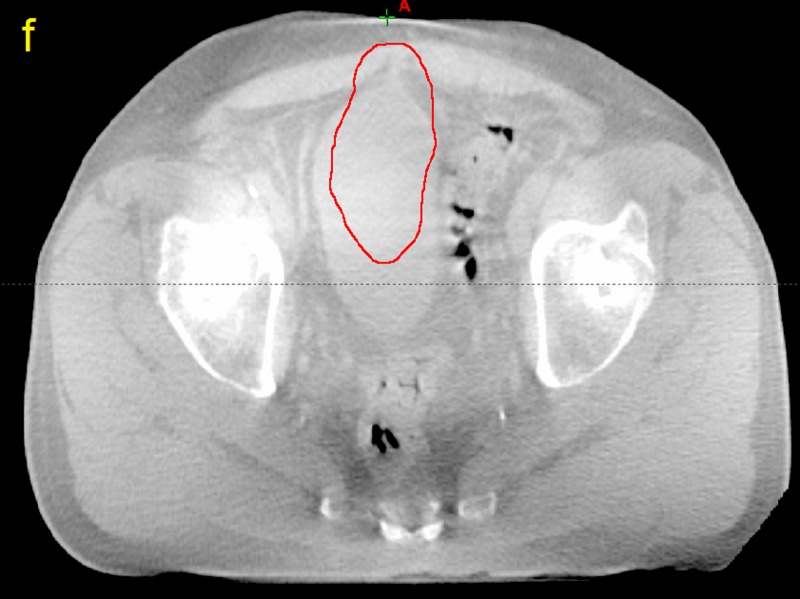
Computed tomographic (CT) image taken from patient P12 f) The planning target volume superimposed on the cone beam computed tomography image

**Figure 7:**
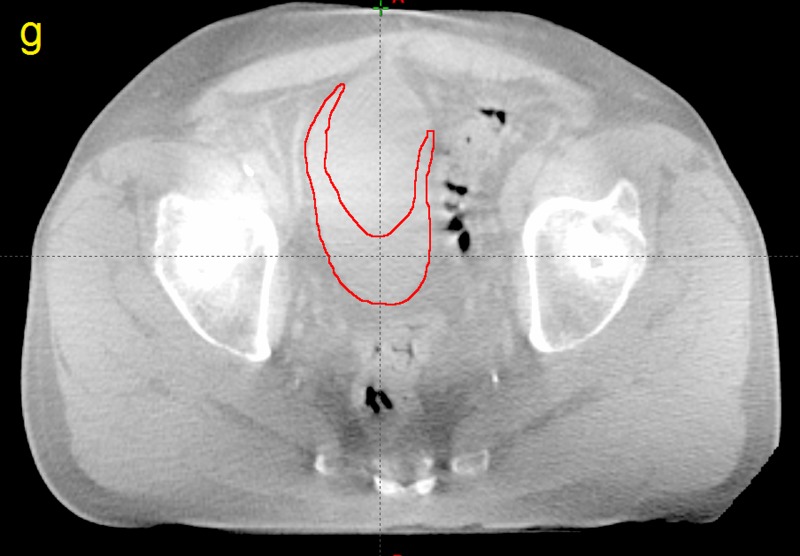
Computed tomography image taken from patient P12 g) The volume of the cone beam computed tomography bladder outside the planning target volume (red)

This study was approved by the University of Manitoba Research Ethics Board. A single observer (AK) retrospectively contoured the bladder CTV on each patient’s planning CT and all CBCTs. A PTV was created by applying anisotropic margins (1.0 cm in the left, right, inferior, and posterior directions and 1.5 cm in the anterior and superior directions). For all contoured bladders, the volume, centre of mass (COM), and the maximal positions (MP) in the anterior, posterior, superior, inferior, right, and left directions were recorded. The volume of bladder outside of the PTV in each CBCT scan was measured (Figures 1-7).

The amount of overlap between each CBCT bladder contour and the patient’s planning CT bladder contour was also measured. The similarity between each CBCT bladder contour and planning CT bladder contour was evaluated using the Dice similarity coefficient (DSC). The DSC is calculated by dividing two times the overlapping volume of two structures by the sum of the two structures’ individual volumes [8]. The mean and standard deviation were calculated for all the bladder outcomes. Eleven outcome variables, including volume ratio (CBCT:planning CT), COM shifts (CBCT relative to planning CT), MP shifts (CBCT relative to planning CT), and DSC in each CBCT were analysed in order to determine if any time trends were present or if there were any relationships between the outcome variables and the patient age, gender, BMI, or disease stage. Random intercept mixed models were used to analyse outcomes: linear mixed models when the assumption of normality was met and linear quantile mixed models when the assumption of normality was not met. Mixed models were used to account for the repeated measures of outcomes. Diagnostics of regression models were performed using residual plots and influence plots. All models included the fraction number as a predictor, and any additional predictors were included using likelihood ratio testing. Linear mixed models were run using PROC MIXED in SAS, v. 9.2 (SAS Institute Inc., Cary, NC, USA) and linear quantile mixed models were run using the R package Linear Quantile Mixed Models (lqmm), version 3.2.0.

The bladder MP data was used to calculate PTV margins using a simplified version of the equation previously described by Meijer, et al. [9]. In our implementation, margins were only calculated in the superior, inferior, anterior, posterior, right, and left directions and not across the entire bladder surface as was done by Meijer, et al. [9]. The evaluated margins account for uncertainties associated with changes in bladder size/shape as well as any residual setup error. The components of the margins due to residual patient set-up error were analysed via the method described by van Herk, et al. [10]. To do this, each CBCT image was re-registered to the planning CT image using Eclipse treatment planning software. Translational setup errors in the x, y, and z aspects that resulted in alignment of the pelvic bony anatomy were recorded. Rotational errors were not assessed as they were assumed to be small [11-12]. 

## Results

A total of 34 patients were initially assessed for inclusion in the study. However, three of the patients did not receive CBCT scans, two patients were excluded as the field of view (FOV) in all CBCT images did not include the inferior aspect of the bladder, and one patient was excluded because of hip prostheses, which caused severe artifacts precluding contouring on CBCT. Hence, 28 patients were included in our study (Table 1).

**Table 1:** Overview of Patient Characteristics

Category	Variable	Number of Patients	Percentage of Patients
Number of Phases	1	14	50.00
	2	13	46.43
	3	1	3.57
Dose(cGy)/Fraction Number	3000/12	1	3.57
	3250/13	1	3.57
	3750/15	1	3.57
	4750/19	1	3.57
	5000/20	8	28.57
	6480/36	16	57.14
Gender	Male	22	78.57
	Female	6	21.43
Disease Stage	I	3	10.71
	II	16	57.14
	III	5	17.86
	IV	4	14.29
Age	50-59	1	3.57
	60-69	6	21.43
	70-79	12	42.86
	80-89	2	7.14
	90-99	7	25.00
Body Mass Index	unknown	2	7.14
	20-24.9	8	28.57
	25-29.9	11	39.29
	30+	7	25.00

Each patient received 12-36 daily fractions of radiation resulting in total doses between 3,000 and 6,480 cGy and three to 11 CBCT scans.

**Figure 8:**
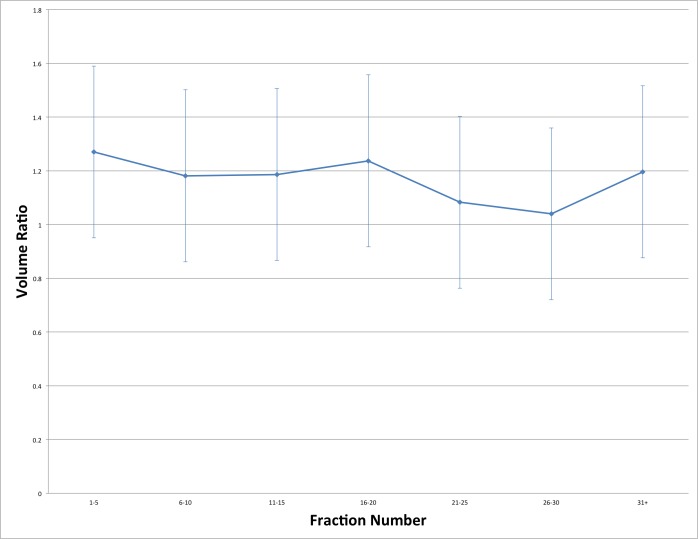
Cone beam computed tomography (CBCT): Planning CT bladder volume ratio as a function of fraction number

The mean +/- standard deviation bladder volume in all scans combined was highly variable at 154.2 +/- 129.4 cm^3^. Overall, the average ratio of CBCT bladder volumes to planning CT bladder volumes was 1.20 +/- 0.32 cm^3^. There was no significant trend in bladder volume ratios with respect to fraction number (Figure 8). 

Table 2 shows the mean shift and standard deviations for bladder parameters. 

**Table 2:** Overview of the Results for the Bladder Outcomes Examined in this Study * The median difference was determined for the volume ratio, superior, and inferior shifts using a linear quantile mixed model. The mean difference was found for all other variables using a linear mixed model. CBCT fraction number used in the models is the original fraction number divided by 10. This was done as the estimates were very small using the original scale. The estimates can be interpreted as “For every 10 fraction numbers, the mean/median outcome will change by _____ units." †: For both the COM and MP shift values, shifts in the x (lateral) component positive values are shifts to the left and negative values are shifts to the right; for the y  (AP) component positive values are shifts anteriorly and negative values are shifts posteriorly; for the z (SI) component positive shifts are inferiorly and negative shifts are superiorly. AP: anteroposterior; CBCT: cone beam computed tomography; COM: centre of mass; MP: maximal positions; SI: superior/inferior

Bladder Parameter		Mean	Standard Deviation	Mean/Median Difference*	p value
Volume Ratio		1.20	0.32	0.013	0.763
Maximal Position Shift (cm)†	Superior	-0.01	0.65	0.108	0.143
	Inferior	0.06	0.14	<0.001	1.00
	Anterior	-0.17	0.48	-0.039	0.245
	Posterior	-0.16	0.60	0.107	<0.001
	Left	0.01	0.34	-0.075	<0.001
	Right	0.02	0.46	0.114	<0.001
Centre of Mass Shift (cm)†	x	-0.05	0.17	0.002	0.877
	y	-0.07	0.42	0.040	0.076
	z	-0.11	0.37	0.045	0.037
Dice Similarity Coefficient		0.75	0.08	-0.017	<0.001

**Figure 9:**
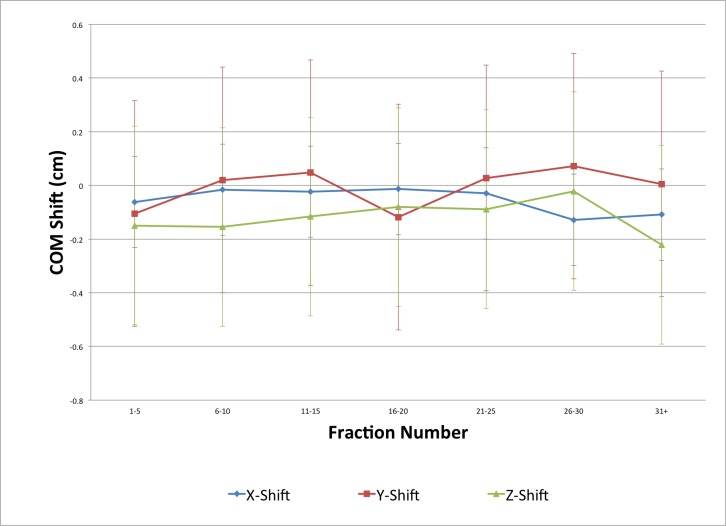
Bladder centre of mass shifts as a function of fraction number

**Figure 10:**
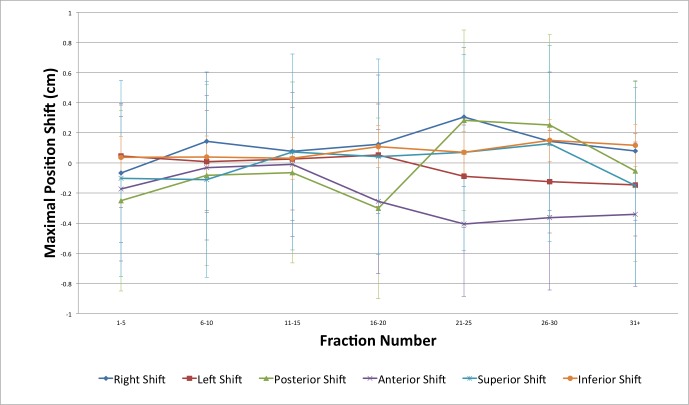
Bladder maximal position shifts as a function of fraction number

Trends with respect to fraction number were statistically significant for the following COM and MP parameters: z-shift (0.05 cm/10 fractions), right shift (0.11 cm/10 fractions), left shift (-0.08 cm/10 fractions), and posterior shift (0.11 cm/10 fractions) (Figures 9-10).

**Figure 11:**
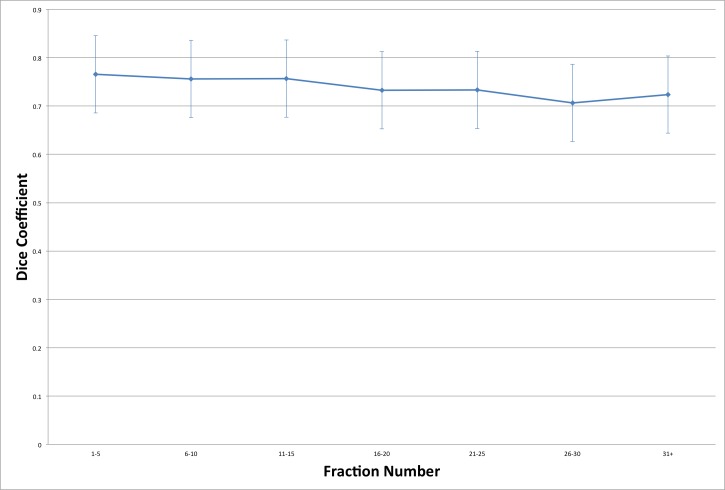
Dice similarity coefficient as a function of fraction number

For the overall patient population, the mean +/- standard deviation DSC was 0.75 +/- 0.08. There was a significant time trend for DSC with respect to fraction number (-0.02/10 fractions) (Figure 11).

Age was shown to be a significant factor for COM z-shift (0.15 cm/10 years, p = 0.029) and superior MP shift (0.30 cm/10 years, p = 0.036). No other statistically significant relationships with patient factors were seen.

**Figure 12:**
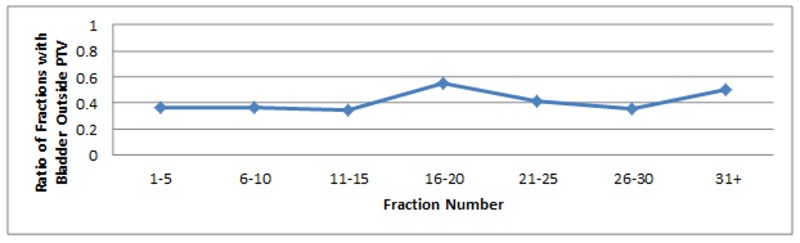
Ratio of fractions with > 0 cm3 of bladder outside of the PTV (compared to total number of fractions) as a function of fraction number PTV: planning target volume

There were 22 out of 28 patients who had at least one fraction in which part of the bladder was not contained within the PTV. Overall, 84 of 212 fractions (39.6%) had at least some of the bladder outside the PTV. However, only 15 of 212 fractions (7.1%) had greater than 10 cm^3^ of CTV outside PTV, 26 of 212 (12.3%) had greater than 5 cm^3^, and 46 of 212 (21.7%) had greater than 1 cm^3^. The mean volume of bladder outside the PTV for all 212 fractions studied was 2.28 cm^3^, with a median of 0.00 cm^3^ and an upper quartile of 0.61 cm^3^ (Figure 12). 

Using a simplified version of the equation previously described by Meijer, et al. [9], the required PTV margins to ensure that 90% of the population receives at least 95% of the prescribed dose are 1.48 cm right, 1.15 cm left, 2.13 cm posterior, 1.52 cm anterior, 2.23 cm superior, and 0.52 cm inferior. 

## Discussion

This study demonstrated the substantial variability in bladder volumes during the course of radiotherapy with no significant trend over time. Large deviations in COM and MP were seen, but only small mean shifts were observed. For the most part, it was determined that there were no statistically significant relationships between the outcomes and the patient factors. Therefore, patient factors cannot be used to clinically predict changes in the outcomes studied. The variability in bladder volume and position seen in this study resulted in a high percentage of fractions in which part of the bladder was outside the PTV.

Other studies have found that bladder volume decreases over the course of radiotherapy, which is in contrast to the present study [11-13]. Our results show a marked fraction-to-fraction volume variation but no statistically significant change in bladder volume over time. Several other groups have described large variations in bladder volume over the course of treatment [14-15]. The lack of significant time trend for bladder volume with respect to fraction number in this study mirrors the results of Turner, et al. and Mangar, et al. [16-17].

It has been suggested that bladder volume decreases over the course of treatment due to a variety of factors, including the development of a consistent bladder emptying routine prior to treatment, the anti-tumour effects of radiation (leading to improved bladder function and, thus, lower residual urine), and increased urgency secondary to radiation induced cystitis [13]. 

Several other groups have attempted to quantify the changes in bladder position over the course of radiation treatment. These studies generally show that the change in bladder position is greatest superiorly and in the AP plane (both anteriorly and posteriorly) [3, 9, 11, 13, 15-16, 18-19]. In terms of MP shifts in this study, the greatest variation was in the anterior (standard deviation (SD) 0.48 cm), posterior (SD 0.60 cm), and superior positions (SD 0.65 cm). The greatest variation in COM coordinates was in the y (anteroposterior or AP) plane (SD 0.43 cm) and z (superior/inferior or SI) plane (SD 0.37 cm). While the variability in these shifts was large, the mean shifts were small because the shifts occurred in both positive and negative directions. The greatest mean shifts observed were in the z COM (-0.11 cm), anterior MP (-0.17 cm), and posterior MP (-0.16 cm) directions. The absence of a large mean shift in the superior direction (as seen in previous studies) [3, 9, 11, 16], while also observing the greatest amount of variability, may be due to the methods used in this study. First, superiorly (and inferiorly), the accuracy of the position measurement was limited by the slice thickness of the CT scans (0.3 cm). Hence, each shift measured had to be in multiples of 0.3 cm. Additionally, in some CBCT scans, artifacts from bowel gas obscured the outline of the bladder superiorly making contouring difficult. Other groups have reported similar difficulties [14]. In the inferior direction for male patients, accurately and consistently contouring the bladder region where it begins to transition to the prostate is difficult [9]. We believe we minimized the uncertainty associated with this by using a single observer for all contours. Statistically significant time trends for changes in the right MP, left MP, posterior MP, and z COM variables relative to fraction number were also observed. However, while statistically significant, the magnitude of these trends was quite small (0.11 cm, -0.08 cm, 0.11 cm, and 0.05 cm per 10 fractions, respectively), and thus these findings are likely not clinically significant.

**Figure 13:**
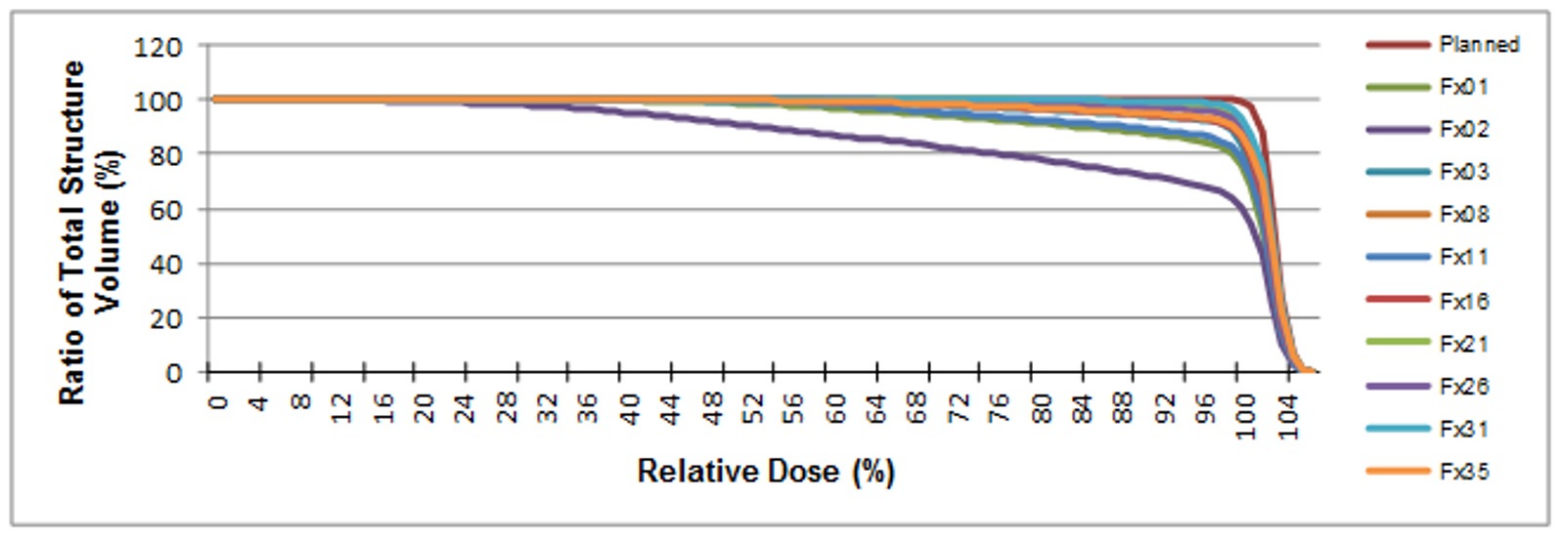
An illustrative dose volume histogram generated for patient P12. The y-axis represents the amount of bladder that received the indicated amount of radiation. Radiation dose is given as a percentage of the prescription dose. Note fraction 2 in which a large volume of bladder was missed due to shifts in bladder volume/position (see Figure 1 for an illustration of the bladder shift)

The amount of bladder volume outside the PTV represents tissue that was supposed to be treated but was missed due to changes in bladder volume and position as well as any residual patient setup errors. The dosimetric effects of these variations are plotted for patient P12 in Figure 13.

Previous studies on this outcome have shown diverse results. Using 1.0 - 2.0 cm isotropic margins, Harris, et al. showed that only two of 20 patients had the bladder expand outside the PTV [4]; Henry, et al. stated the CTV remained within the PTV 93.5% of the time using 1.5 cm isotropic margins [14]. However, Pos, et al. claimed 65% of patients had at least one fraction with CTV outside the PTV using 1.5 - 2.0 cm isotropic margins [20]. Yee, et al. measured the average volume outside the PTV to be 2.41 cm^3^ with 1.0 - 1.5 cm isotropic margins [13]. This study’s results are more in keeping with those of Yee and Pos. Table 3 contains a summary of all studies discussed, which also examined bladder motion and how it impacted radiation therapy for muscle invasive bladder cancer. 

**Table 3:** Summary of Studies Examining Bladder Motion During Radiotherapy CBCT: cone beam computed tomography; CT: computed tomography; ITV: internal target volume; PTV: planning target volume; SD: standard deviation

Authors	Year Published	Radiation Dose	Results
Fokdal, et al. [3]	2004	60 Gy	- The internal margins required to cover the bladder movements in 87% of the patients were 2.4 cm in the anterior, 1.1 cm in the posterior, 3.5 cm in the cranial, 0.5 cm in the caudal, and 1.3 cm in the lateral direction.
Harris, et al. [4]	1998	60 Gy	- The bladder dome rose out of the treatment field in two patients during the course of therapy. - In 16 patients, the target volume was encompassed as planned throughout.
Meijer, et al. [9]	2003	60 Gy	- Organ motion is the predominant geometric uncertainty in the radiotherapy process (5 mm, 1 SD, at the cranial side of the bladder).
Muren, et al. [11]	2003	60 – 64 Gy	- Repeat scan bladder volumes extended outside the planning scan bladder contours in 89% of the scans, on average with 9% of the volume (range: 0 – 47%). - CTV-to-ITV margins of 10 mm inferior, 20 mm superior, 11 mm left, 8 mm right, 20 mm anterior, and 14 mm posterior were required to simultaneously encompass all bladder deflections, except for the largest outward deflection in all directions in 84% of the patients.
Foroudi, et al. [12]	2013	64 Gy	- Required margins to cover intrafraction changes from pretreatment to post treatment in the superior, inferior, right, left, anterior, and posterior were 1.25 cm (range, 1.19-1.50 cm), 0.67 cm (range, 0.58-1.12 cm), 0.74 cm (range, 0.59-0.94 cm), 0.73 cm (range, 0.51-1.00 cm), 1.20 cm (range, 0.85-1.32 cm), and 0.86 cm (range, 0.73-0.99), respectively.
Yee, et al. [13]	2010	50 - 65 Gy	- Mean CBCT PTV outside the planning CT-derived PTV was 47.35 cm^3^ (SD, 36.51 cm^3^). - Mean planning CT-derived PTV outside the CBCT-derived PTV was 93.16 cm^3^ (SD, 50.21). - Mean CBCT-derived bladder volume outside the planning PTV was 2.41 cm^3^ (SD, 3.97 cm^3^).
Henry, et al. [14]	2006	47.5 – 55 Gy	- 93.5% of imaged fractions, the clinical target volume was within the planning target volume.
Lotz, et al. [15]	2006	55 – 60 Gy	- Gross tumour volume translations were largest in cranial–caudal and anterior–posterior direction (SD, 0.1 to ~ 0.9 cm).
Mangar, et al. [16]	2008	Not stated	- Mean weekly variation in bladder volume relative to the planning volume was 0-12% (standard deviation 20-34%) with no observable trends over time. - Regression analysis showed that it is possible to ensure complete coverage of the bladder with a 1 cm margin, providing the volume did not exceed over 50% of the initial planning scan volume.
Turner, et al. [17]	1997	45 – 52.5 Gy	- 18 of 30 patients (60%) demonstrated "significant" movement of at least one bladder wall relative to the original isodose plot. - Movement resulting in margin reduction occurred in 10 patients (33%).
Sur, et al. [18]	1993	48 – 55 Gy	- 72 patients (80%) had no spatial shift in the target volume, but of the 18 patients with such a shift, treatment plans were changed in seven.
Burridge, et al. [19]	2006	52.5 Gy	- Bladder of 1 patient was systematically smaller than the planning scan and hence demonstrated the largest average reduction of 76 cm^3^. - The clinical target volume to PTV margins in other directions can be safely reduced to 10 mm except in the anterior direction where, like the superior direction, the bladder showed significant variation.
Pos, et al. [20]	2003	60 Gy	- In 65% of patients, a part of the tumour appeared outside the planning target volume boundaries at least one time during the course of radiotherapy.

Various patient factors (age, gender, BMI, and disease stage) were also examined to see if there were any relationships with changes in bladder volume, COM, MP, or DSC. Previously, no associations between bladder volume/position and age, body size, or tumour stage were found [17]. The results of this analysis showed that age had a statistically significant relationship with the z and superior shifts, meaning for every 10 years of age, the mean z shift and the median superior shift will change by 0.15 cm and 0.30 cm, respectively. Since the magnitude of these relationships is quite small and the lack of statistically significant relationships with other bladder parameters, we do not feel that there are any patient specific factors, which can be used clinically to predict changes in bladder position and/or volume over the course of radiation therapy.

The effect of patient setup error was small and had much less impact on the margins compared to changes in bladder volume/position (margins required for residual patient setup error alone were 0.32 cm right, 0.32 cm left, 0.47 cm posterior, 0.47 cm anterior, 0.43 cm superior, and 0.43 cm inferior). It is worth noting that our posterior margin value (2.13 cm) calculated in this study is much larger than the values previously proposed [3, 11]. It is not clear why such large shifts of the bladder posteriorly compared to the planning CT scan were seen. One possibility could be decreased rectal filling as this has been shown to cause posterior shifts of the bladder [3].

Potential options for mitigating the impact of bladder volume changes, other than increasing PTV margins, include increased diligence in reminding patients to void their bladders prior to treatment, catheterization prior to treatment [3, 14], medical optimization of conditions (such as benign prostatic hypertrophy (BPH) which may impair bladder emptying) [14], and treatment of co-existing cystitis. To mitigate the effect of differing rectal volumes on bladder position, one could consider laxatives, a low-residue diet, or insertion of a rectal balloon to achieve more consistent rectal filling. It should be noted that the degree of rectal filling has less impact on bladder volume and position than bladder filling so the effect of rectal interventions may be modest [3].

A different approach to this issue is a daily adaptive plan selection in which a CBCT is taken prior to each treatment, and based on the bladder size and position, a suitable plan would be chosen for that day [21-22]. An obstacle to this is the additional resources necessary for creating multiple radiotherapy plans. Potential solutions to this problem have been explored. One is using a composite of the planning CT and CBCTs from the first five fractions to create small, medium, and large adaptive plans [22]. Lutkenhaus, et al. described a method in which planning CT scans were taken with a full and empty bladder and various intermediate volumes were interpolated based upon those scans. This allowed the group to create five different treatment plans, which could be chosen from based upon daily CBCT imaging results during treatment [21]. Another simpler solution may be to have a radiation therapist examine the CBCT scan taken prior to treatment. If any (or greater than a mandated amount of) bladder in the CBCT scan is outside of the PTV, they could have the patient void his/her bladder and then return to the treatment unit and redo the CBCT. If over the course of treatment a consistent, clinically significant change in the patient’s bladder volume or position was noted, the treating physician could consider replanning to account for this change. 

One limitation of our data is that seven patients missed at least one of the first three CBCT scans, which may have affected the ability to identify a trend. However, additional CBCTs near the start of treatment were only acquired because of the previous studies, which showed decreased bladder volume over the course of radiotherapy [11-13]. Because this trend was not observed in our patients, the effect of missing those scans was likely minimal. 

## Conclusions

In conclusion, this study showed substantial variation in bladder volume and position over the course of radiotherapy. However, no clinically relevant trends related to fraction number or patient factors could be identified. Because of these seemingly random bladder changes, there were some fractions in which the CTV fell outside of the PTV; however, in most cases, the volume of CTV was small. Increasing margins to account for the days in which a large bladder volume increase occurs is not practical. Instead, other methods to minimize the amount of CTV that is missed on a fraction-to-fraction basis should be explored. 
